# Fibromine is a multi-omics database and mining tool for target discovery in pulmonary fibrosis

**DOI:** 10.1038/s41598-021-01069-w

**Published:** 2021-11-05

**Authors:** Dionysios Fanidis, Panagiotis Moulos, Vassilis Aidinis

**Affiliations:** 1 Institute for Bioinnovation, Biomedical Sciences Research Center ″Alexander Fleming″, 16672 Athens, Greece; 2 Institute for Fundamental Biomedical Research, Biomedical Sciences Research Center ″Alexander Fleming″, 16672 Athens, Greece

**Keywords:** Data integration, Data mining, Databases, Software, RNA sequencing, Transcriptomics, Protein-protein interaction networks

## Abstract

Idiopathic pulmonary fibrosis is a lethal lung fibroproliferative disease with limited therapeutic options. Differential expression profiling of affected sites has been instrumental for involved pathogenetic mechanisms dissection and therapeutic targets discovery. However, there have been limited efforts to comparatively analyse/mine the numerous related publicly available datasets, to fully exploit their potential on the validation/creation of novel research hypotheses. In this context and towards that goal, we present Fibromine, an integrated database and exploration environment comprising of consistently re-analysed, manually curated transcriptomic and proteomic pulmonary fibrosis datasets covering a wide range of experimental designs in both patients and animal models. Fibromine can be accessed via an R Shiny application (http://www.fibromine.com/Fibromine) which offers dynamic data exploration and real-time integration functionalities. Moreover, we introduce a novel benchmarking system based on transcriptomic datasets underlying characteristics, resulting to dataset accreditation aiming to aid the user on dataset selection. Cell specificity of gene expression can be visualised and/or explored in several scRNA-seq datasets, in an effort to link legacy data with this cutting-edge methodology and paving the way to their integration. Several use case examples are presented, that, importantly, can be reproduced on-the-fly by a non-specialist user, the primary target and potential user of this endeavour.

## Introduction

Idiopathic pulmonary fibrosis (IPF) is a chronic, progressive idiopathic pulmonary disease mainly manifested in older adults and characterized by extensive fibrosis of the lung interstitium that irreversibly affects normal lung function^1^. Although presenting a highly heterogeneous clinical course, half of the IPF patients succumb to respiratory failure or life-threatening comorbidities within 2–5 years post diagnosis^2–4^. Its enhanced morbidity, increased incidence among elder individuals^4^ and the lack of a curative option^2^ render IPF a rare disease of major concern, especially for ageing societies. Currently, new studies focus on providing healthcare practitioners with invaluable information about patient stratification and prioritization, non-pharmaceutical treatment options and medications confronting potentially fatal comorbidities^2^. Nevertheless, the task of new disease targets discovery is still greatly unfulfilled, as the most currently approved anti-fibrotics, nintedanib and pirfenidone, may delay lung function decline, but cannot help patients evade a fatal outcome^5^.

Since their first appearance, omics technologies have been extensively used to assess pathological deregulation in multiple molecular levels, thus physically leading to the progressive accumulation of a great volume of publicly available datasets. As far as IPF is concerned, a large number of expression profiling studies in IPF patients, as well as animal models, have been performed providing the scientific society with important lists of implicated targets such as that of Kim et al.^6^. Most of their datasets have been deposited in public repositories, but the qualitative assessment and mining of this vast legacy information, require significant expertise and time investment, not always available at the average research lab.

Irrespective of its laborious nature, comparative meta/re-analysis of publicly available datasets would provide additional means to discover novel pathogenic targets. Towards this direction, several attempts have been made to integrate IPF datasets^7–11^, yet none of them spans a comprehensive collection of sample types, technologies and pathology models, while real-time data exploration features are either limited or absent.

Recognizing the need for a centralized, easily operable and comprehensive source of IPF(-related) data, we here introduce Fibromine, an open source application and database of integrable omics datasets accompanied by rich gene/RNA/protein level annotation and dataset meta-data. Currently, Fibromine hosts 60 consistently re-analysed and manually curated transcriptomic and proteomic datasets ranging over 42 unique comparisons, two species and several cell culture-based experiments. Moreover, and to increase data resolution, our database hosts a collection of more than 200,000 single cells originating from healthy and various pulmonary diseases samples. Fibromine can be accessed through a website (http://www.fibromine.com/Fibromine) that offers, amongst others: (i) within and across species integration of the supported datasets (*Dataset explorer*), (ii) interrogation of coding and non-coding gene expression patterns (Gene explorer; miRNA explorer), and (iii) exploration of protein abundance changes during disease (*Protein explorer*). In addition, other functionalities include the creation of disease-specific protein–protein interaction (PPI) (*Protein explorer*) and lung-specific gene co-expression networks (*Gene co-expression*), inter-connection with single cell resolution data visually via *Gene explorer* and in more detail via *Single cell data* tab, pathway analysis of consensus differentially expressed genes (cDEGs) and creation of heatmaps and volcano plots (*Dataset explorer*). Last but not least, in order to guide the user through transcriptomic datasets’ underlying characteristics and inherent variability, a dataset benchmarking strategy based on seven distinct metrics has been applied (*Datasets benchmarking* tab). Conclusively, Fibromine not only aims to facilitate specialist and non-specialist users to validate their findings/observations and/or form nascent hypotheses prior to any time-consuming wet lab validation, but also to boost downstream research attempts via unconstrained access to all database data and integration/mining outputs.

## Results

### Fibromine datasets selection, re-analysis, curation and organization

**Figure 1:**
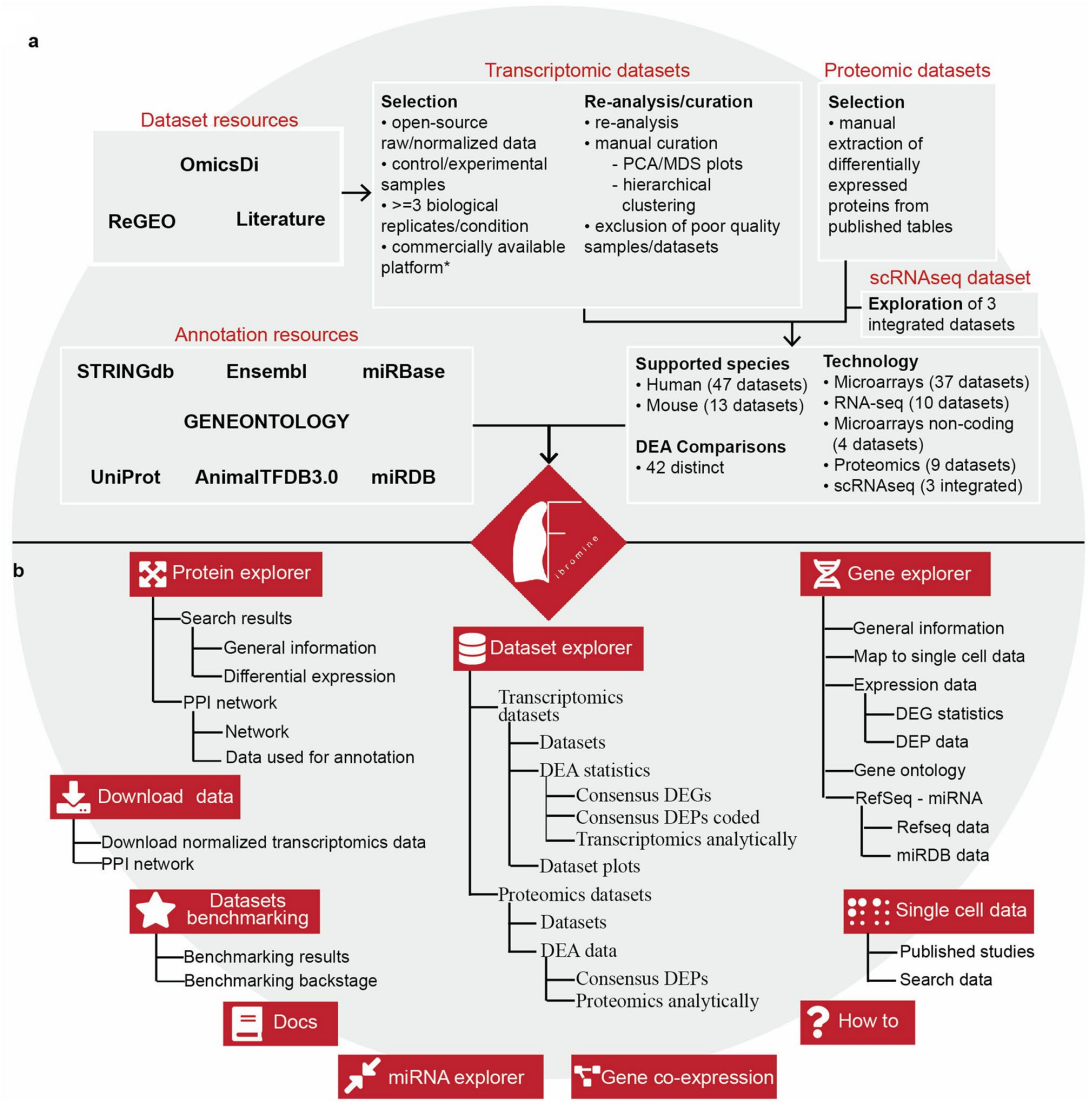
Fibromine database creation workflow and homonymous web server map. **(a)** Fibromine included datasets were discovered via literature, https://www.omicsdi.org/ and http://www.regeo.org/ scrutiny prior to specific criteria-based selection. Transcriptomic datasets were re-analysed and manually curated, while differentially expressed proteins were gleaned from respective publications. Differential expression was performed in a collection of three integrated scRNA-seq datasets. Annotation was sourced from various open-access resources. **(b)** Fibromine web interface is Shiny-powered. It is made out of three main explorers *Dataset*, *Gene* and *Protein explorer* and several smaller ones, while *Datasets benchmarking* tab provides a metrics-based system of datasets accreditation. *Single cell data* tab provides a list of relative published studies along with DEA results from three integrated datasets. The user can also map a gene directly to several single cell datasets via *Gene explorer*. *MDS:* multi-dimensional scaling, *PCA:* principal component analysis. Annotation sources: https://string-db.org/, https://ensembl.org/, https://mirbase.org/, http://geneontology.org/, https://uniprot.org/, http://bioinfo.life.hust.edu.cn/AnimalTFDB/, http://mirdb.org/. Figure was created using https://www.google.com/slides/about/.

Aiming to create a centralized repository for IPF omics data, we catalogued a plethora of publicly available microarray and RNA-seq transcriptomic datasets via PubMed and omicsdi.org using “IPF” and “bleomycin” as search keywords for human and mouse datasets, respectively. In the case of omicsdi.org, search results were further narrowed down using the “Transcriptomics” filter. Datasets included in the work of Villaseñor-Altamirano^11^ were also taken into consideration and finally, references from an IPF transcriptomics review^3^ along with results from ReGEO^12^ search using the “IPF” keyword were intersected with the already found datasets to form an initial pool of IPF datasets. To maintain the most informative, we excluded, among others, those consisting of less than three biological replicates, those of poor data quality (see further down), as well as those created using custom developed platforms (Fig. 1a). A few exceptions to the latter criterion were made to include datasets such as GSE31934 that address rarely explored scientific questions.

A major issue in all meta-analytic efforts that include data from various technological principles is the presence of technical heterogeneity within and across datasets that can, if ignored, greatly affect the data integration results by introducing non-biological sources of variability. Hence, to address technical variability in a scalable fashion that would facilitate future Fibromine updates without the requirement of already included data re-processing, we have re-analysed the collected transcriptomic datasets in a modular fashion maintaining the same re-analysis methods/parameters both across and within technologies in the highest degree possible. Then, to pinpoint and remove any sample outliers, all re-analysed datasets were manually curated using Principal Component Analysis (PCA)/MultiDimensional Scaling (MDS) and samples hierarchical clustering plots (Fig. 1a, Supplementary Fig. S1). Datasets failing to pass quality control criteria were excluded.

Subsequently, in order to further increase data resolution, we have pinpointed marker genes of all pathology-originating cells compared to healthy donated ones, as integrated by Mayr et al.^13^. Pathology versus healthy state comparisons were performed for over 40 cell types’ top variable genes, resulting in an expression profiling of ten different pulmonary diseases.

Furthermore, in order to complement differential gene expression data, we retrieved deregulated proteins between IPF and control/healthy individuals from respective publications (Fig. 1a). The latter were selected via an extensive manual literature search and the only criterion applied for dataset selection was the provision of Differential Expression Analysis (DEA) results for more than just a couple of proteins in a tabular format. Proteomic datasets selection procedure was not as strict as the one for gene expression data, due to the relatively small number of the former publications relative to the latter^14,15^. Proteins quantified in mixtures/same aptamer were removed, while DEA thresholds were kept *as is* in each original publication.

With the purpose of organising the aforementioned data, we created Fibromine, a dedicated application backed by a manually curated database that holds both DEA results and normalized transcriptomics data from 47 human and 13 murine datasets (Fig. 1a, Supplementary Fig. S2). Subsequently, because several datasets support multiple DE comparisons, we armed Fibromine with a controlled vocabulary of terms so as to be able to effectively codify and match all included phenotypes/DE comparisons across datasets. More specifically, based on respective literature and GEO retrieved nomenclature, all DEA comparisons were denoted by a three-part phrase, such as *A_vs_B* (or *A vs B*), where the first and third elements represent the two participating experimental conditions. Any dashes or underscores used in the comparisons pre-fix and suffix elements, *A* and *B*, separate sample from potential treatments and/or time-points. The capital letter *D* followed by one or multiple digits is used to denote the day of sample collection respective to the starting time-point of a given treatment.

Last, to enrich these data and support an unhampered by external parameters data exploration experience we have also included into Fibromine gene, transcript and protein level annotation retrieved from Ensembl^16^, UniProt/Swiss-Prot^17^ and STRING databases^18^, as well as gene ontology terms from Refs.^19,20^, miRNA annotation downloaded from mirBase^21^ and miRNA target interaction predictions from miRDB^22^ (Fig. 1a). Human and mouse transcription factor annotation was obtained from Refs.^23,24^, respectively. All operations were supported by in-house developed R/bash scripts and the use of GEOquery^25^ and biomaRt^26^ Bioconductor packages.

Conclusively, Fibromine is an ab initio created IPF-oriented database that interlinks a great volume of DEA results with rich out-sourced annotation. In addition, it is worth mentioning that Fibromine is the first lung fibrosis database that encompasses multi-omics and multi-species data, thus offering a great starting line for comparative meta-analyses.

### Fibromine datasets benchmarking

**Figure 2:**
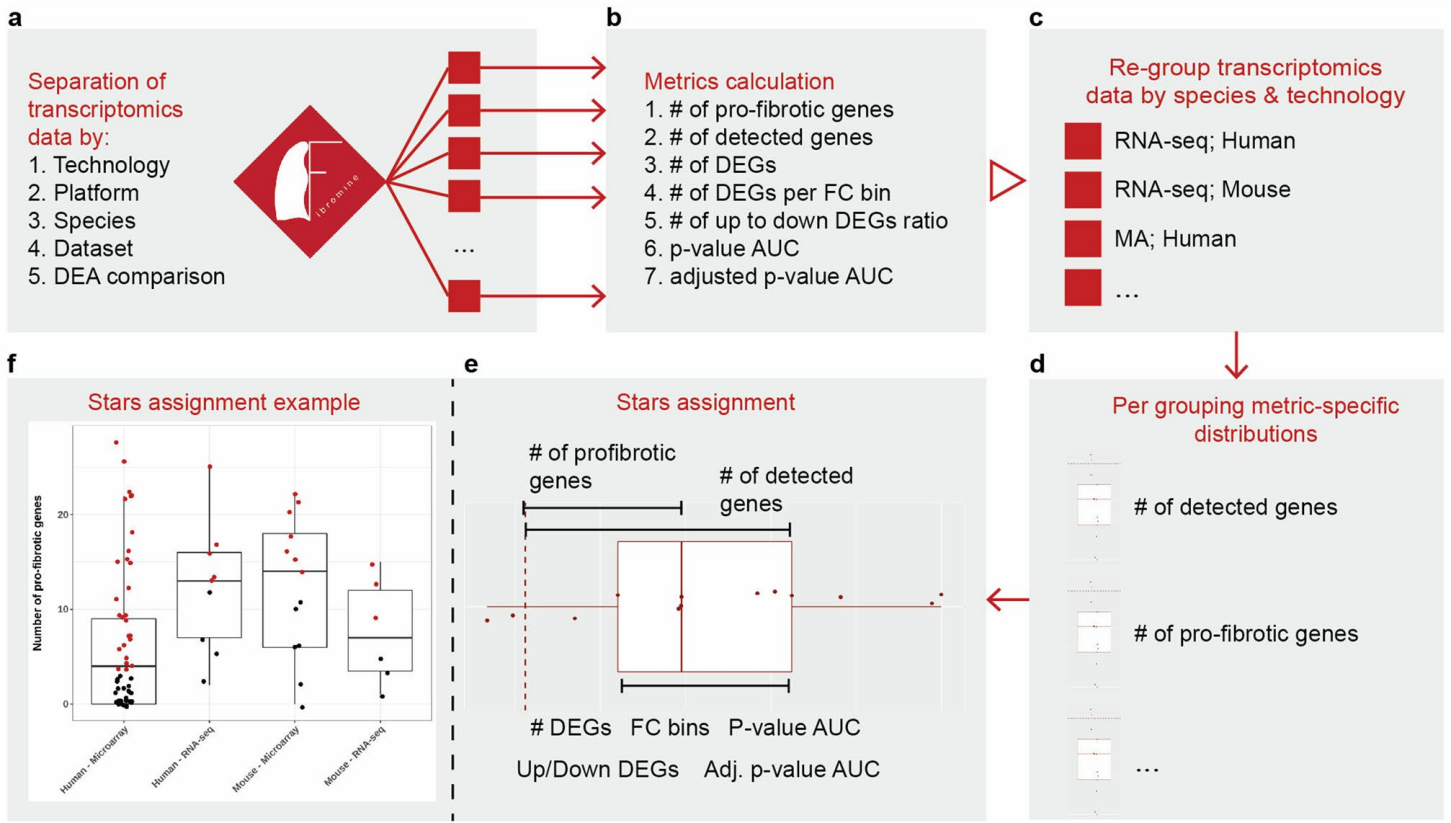
Fibromine transcriptomic datasets benchmarking workflow. In order to reveal the most homogeneous transcriptomic datasets of Fibromine, seven metrics were calculated **(b)** post to data separation **(a)**. Subsequently, transcriptomic data were grouped per species and technology **(c)** and seven metric-specific distributions were shaped for each of the dataset groups **(d)**. Finally, every dataset/DEA comparison was evaluated relative to the rest of its grouping and assigned a star if the calculated criterion value lied within a pre-specified interval of the respective distribution **(e)**. Each dataset received 7 stars to the maximum and none to the minimum. Datasets with many stars are more closely related to each other than to the rest of the group. **(f)** A real example of stars assignment: red dots are datasets/DEA comparisons assigned a star for the *Number of pro-fibrotic genes* criterion. Datasets investigating exclusively non-protein coding genes were processed separately using the same workflow. Boxplots depict the interquartile range and median of the data; whiskers extend no longer than 1.5 times the length of the boxplot. *DEGs:* differentially expressed genes, *FC:* fold change, *AUC:* area under the curve. Figure was created using https://ggplot2.tidyverse.org/ v3.5.5 and https://www.google.com/slides/about/.

As meta-analytic collections are prone to factors introducing biological/technical heterogeneity and IPF is a highly heterogeneous pathology, Fibromine’s controlled vocabulary may be up to a certain point capable of standardizing the DE comparisons included, but definitively cannot assess datasets underlying biological and technical differences that may affect any downstream integration attempt. For this reason, as well as the fact that most datasets’ meta-data do not comprehensively describe crucial information that would assist in a variability reduction endeavour, instead of directly dealing with data variability we next set to offer a thorough means of datasets evaluation, easily interpretable by all users. For such a purpose, we designed a dataset assessment system, which we implemented on dataset/DEA groupings (Fig. 2a) in order to distinguish and accredit the most similar datasets within certain groups shaped using experimentally-based criteria. Our strategy is currently based on seven broadly used metrics: (i) the number of detected and (ii) differentially expressed genes, (iii) the representation of known pro-fibrotic genes among each dataset’s Differentially Expressed (DE) genes, (iv) the number of genes with low {x, 1.2 < x < 2)}, intermediate {x, 2 ≤ x < 5} or high {x, 5 ≤ x} absolute fold change, (v) the ratio of up- to down-regulated genes, as well as the area under their (vi) nominal and (vii) adjusted p-value distributions. Human pro-fibrotic genes already known to be implicated in pulmonary fibrosis were collected both from Ref.^3^ and from our lab’s multi-year observations, while their homologue counterparts, as reported in Ensembl database, were used for murine datasets assessment.

More specifically, we separated all bulk transcriptomic data by technology, platform, species, dataset and DEA comparison and then calculated the aforementioned metrics (Fig. 2a,b). Afterwards, data were grouped per species/technology and metric-specific distributions were constructed (Fig. 2c,d). Last, datasets/DEA comparisons were accredited a star for every calculated value of a metric lying within a pre-specified distribution interval (7 stars at maximum) (Fig. 2e,f, Supplementary Fig. S3). All benchmarking results are recorded at *Dataset* and *Gene explorer* matrices, while an analytical description of the process along with intermediate products can be found at *Datasets benchmarking* tab of Fibromine. Datasets/DEA comparisons with more stars are most similar to each other relative to the rest of the group. Datasets investigating exclusively non-coding genes were processed separately using the same workflow. Finally, because our database addresses a wide variety of experimental conditions which are expected to affect benchmarking results when assessed simultaneously, we have repeated the aforementioned process exclusively for datasets belonging to important Fibromine comparisons (*IPF_vs_Ctrl* and *BleomD14_vs_Ctrl* on lung tissue) (Supplementary Fig. S4).

To conclude with, although not completely treating data variability, the realized datasets benchmarking strategy provides an extra layer of datasets comparative information, not readily available neither from raw, publicly available data, nor directly extractable from respective publications, based on which the most interesting/ “homogeneous” datasets can be picked for integration and/or mining.

### Fibromine online interface: a web tool for IPF data integration and mining

A central pillar of the hereby described endeavour, is to facilitate data mining (mainly data summarization and aggregation) and comparative exploration of Fibromine data for the non-specialist user. For this reason, we have developed a Shiny-based online application that enables data access from three distinct but complementary points of observation: from a dataset (*Dataset explorer*), from a gene (*Gene explorer; miRNA explorer*) and from a protein level (*Protein explorer*). In addition to the aforementioned layers of information, *Gene co-expression* tab graphically displays significant modules of human and mouse lung-specific gene co-expression networks, while *Single cell data* > *Search data* tab presents in a tabular format DEA results for a great number of single cells.

### Dataset explorer

**Figure 3:**
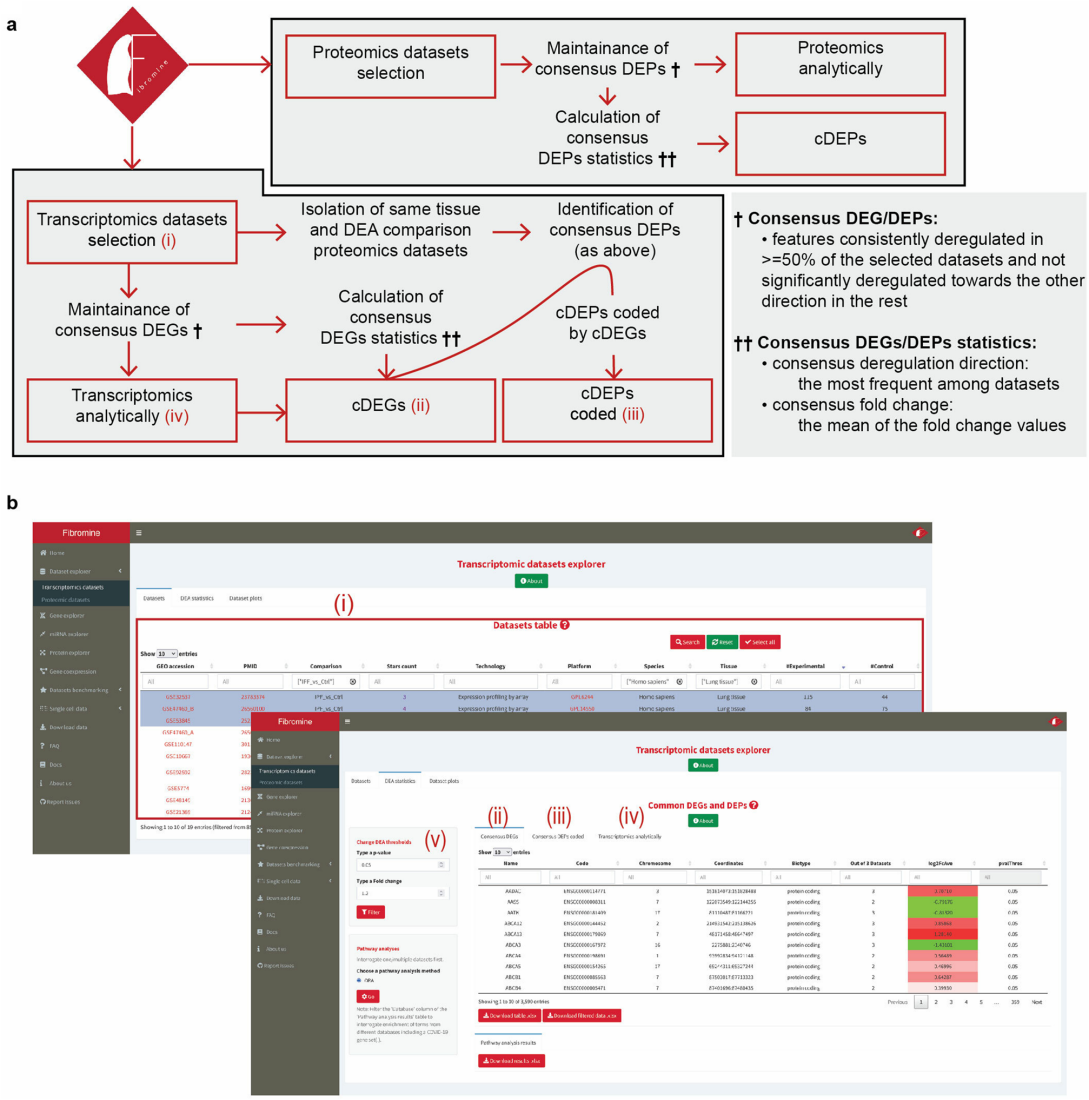
Same species datasets integration workflow. **(a)** *Dataset explorer* same species transcriptomic/proteomic datasets integration workflow. Boxes of red correspond to front-end elements, while the rest account for back-end processes. Latin number elements are presented in **(b)**. **(b)** Front end steps of transcriptomic datasets integration through *Dataset explorer*: (i) datasets selection, (ii) consensus DEGs table, (iii) consensus DEPs coded by any of the consensus DEGs and sharing the same direction of deregulation, (iv) analytical statistics table. Differential expression default parameters can be changed via a dedicated tuning panel (v). *lof2FcAve* column of (ii) holds the consensus direction of deregulation for each of the reported genes in a color coded fashion. *Out of … Datasets* column of the same table enables further tuning of consensus features identification procedure. *cDEGs:* consensus differentially expressed genes, *cDEPs:* consensus differentially expressed proteins. Figure was created using https://www.google.com/slides/about/.

To begin with, Fibromine’s *Dataset explorer* is divided into two tabs responsible for bulk transcriptomic (*Transcriptomic datasets*) and proteomic datasets (*Proteomic datasets*) (Fig. 1b), each one organised around a central interactive table. The user’s input is minimized to the selection of one (exploration) or multiple datasets of interest (integration) and the subsequent press of a button. Particularly, as far as transcriptomic datasets integration is concerned, Fibromine initially detects between datasets cDEGs (Fig. 3, Supplementary Fig. S5). Subsequently, it examines the available proteomic datasets matching the selected datasets experimental parameters for any consensus differentially expressed protein (cDEP) coded by any of the cDEGs. Addedly, in order to facilitate biological interpretation of the cDEG list, the user can perform over-representation analysis based on five gene-term libraries, exploiting the *Pathway analyses* tool provided. Concerning proteomic datasets integration, cDEPs are identified using the same pipeline of cDEGs determination. For more technical details about cDEGs and cDEPs, please, refer to "Methods" section of the paper.

Understanding the limitations and subjective nature of adopting strict statistical thresholds for consensus differentially expressed features (cDEFs) definition (“Methods” section), we have enabled the user to adjust the default fold change and hypothesis testing constraints used during transcriptomics data integration. This feature is not available for proteomic datasets, because the original publication thresholds were considered *as is* for the identification and isolation of Fibromine-included DE proteins. Moreover, the user can further influence the strictness of cDEFs report via the *Out of … Datasets* column of *Consensus DEGs/Consensus DEPs* result tables, that controls the number of datasets out of the n user-selected ones where a feature’s expression has to be found consistently divergent, in order to be recognised as a cDEF (“Methods” section).

Last, as an extra means for transcriptomic datasets inspection, an interactive exploratory heatmap and volcano plot can be depicted upon demand at the *Dataset plots* tab of the explorer. There, the user can examine samples hierarchical clustering and create a volcano plot for each queried dataset. Selected markers of fibrosis are shown in the volcano plot providing a coarse-grained estimation of each dataset’s quality.

### Gene explorer

Being able to acquire differential gene expression data across a great number of samples and datasets in a targeted fashion, is a catalytic step towards supporting wet lab findings and fuelling novel hypotheses formation. To painlessly retrieve the aforementioned information from Fibromine, we have implemented *Gene explorer* (Fig. 1b, Supplementary Fig. S6). Taking as input one or multiple genes of interest, *Gene explorer* displays the statistics (fold change, nominal and FDR adjusted p-value) of all their DE instances found in the database, along with information about their genomic position and biotype, related GO terms and RefSeq sequences. Moreover, as several miRNAs have been reported to be implicated in IPF pathology^9^, the display of miRDB-sourced mRNA-miRNA interactions relative to the queried protein-/miRNA-coding gene at the latest tab of the explorer can prove of extreme use. In parallel, the explorer displays data for any DEP coded by the queried genes. Last, in order to combine the better established methods of bulk DEA with the higher resolution of single cell data, *Gene explorer* maps each requested gene to single cell datasets of NU-Pulmonary cell browser in a species specific manner: human to Reyfman^27^ and murine to Joshi-Watanabe^28^ and Xie^29^ datasets. More details on published scRNA-seq pulmonary fibrosis datasets can be found in the *Single cell data* tab of our web server, along with DEA results of Mayr et al. dataset^13^ in a tabular format. Please, refer to "Single cell data" section of the paper for more details.

### miRNA explorer

Among the transcriptomic studies hosted by Fibromine, there are some having explicitly sequenced non-coding genes. Although these transcripts and their potential targets can be individually interrogated via *Gene explorer*, the latter does not simultaneously provide differential expression statistics for both coding and non-coding interactors. Thus, in order to accelerate the discovery of important regulatory interactions, we have created *miRNA explorer* (Supplementary Fig. S7). In more detail, we have integrated all *IPF_vs_Ctrl* lung non-coding array datasets and isolated the consensus differentially expressed miRNAs. Subsequently, we have performed likewise for their miRDB-sourced targets found at the bulk *IPF_vs_Ctrl* lung coding datasets. Both integrations were performed using the same pipeline applied to define cDEFs (“Methods” section) and |FC|> 1.2 and FDR < 0.05 values were used as differential expression thresholds. As a result, the user can mine the most important regulatory pathways taking place during lung fibrosis by selecting any member of the deregulated miRNA list and automatically obtain all mRNA targets characterized by an opposite direction of expression.

### Protein explorer

**Figure 4:**
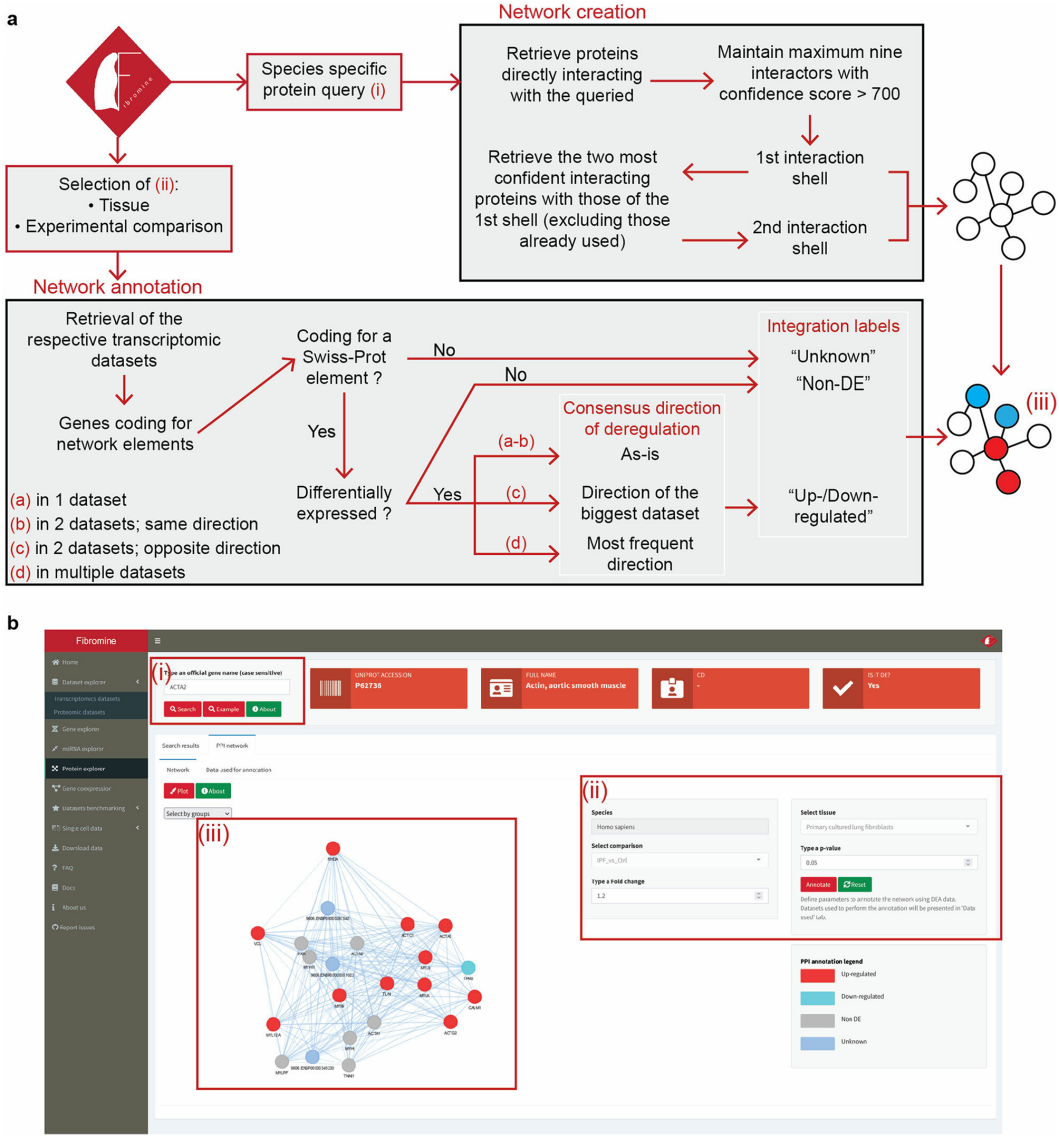
Protein–protein interaction network creation and condition-specific annotation workflow. **(a)** *Protein explorer* back-end process for the creation and annotation of protein–protein interaction networks. Data on protein relationship and interactions confidence are retrieved from Fibromine included UniProt data. Boxed and Latin numbers in red correspond to front-end elements presented in **(b)**. **(b)** The pipeline on the front-end of *Protein explorer*: (i) specific protein query, (ii) selection of experimental parameters to consider, (iii) annotated network. Figure was created using https://www.google.com/slides/about/.

Although the IPF related proteomic datasets are significantly fewer than the respective transcriptomic ones^14^, the proteomic milieu may hold key leads for deciphering IPF progression. For this reason, we have equipped Fibromine with *Protein explorer* (Fig. 1b), a proteomic datasets accessor and protein–protein interaction network creation tool. Requiring as input only a protein’s coding gene name, the explorer initially presents some basic annotation for the queried protein, alongside the respective differential expression data, if any. Subsequently, aiming to inter-connect transcriptomic and proteomic expression data, the explorer hosts a condition-specific PPI network creation tool under the homonymous tab (Fig. 4). With the push of a button, the user can in real-time create a two-shell, high-confidence interactive network revolving around the queried protein and then automatically annotate it upon demand, based on the expression pattern of its nodes in any Fibromine-supported transcriptomic dataset/experimental comparison.

As far as network annotation is concerned, DE statistics for all genes coding for the UniProt/Swiss-Prot members of the network are initially isolated from the datasets corresponding to the experimental parameters selected by the user. Then, differential expression data are summarized for each of the genes into integration labels holding the consensus direction of deregulation: *Non-DE, Up-regulated* and *Down-regulated*. Genes coding for TrEMBL proteins are assigned the *Unknown* label as Fibromine does not currently support TrEMBL entries. The aforementioned labels are used to color-code network nodes and thus transfer all available transcriptomics information into the protein level. Analytical statistics of the data used to annotate the network can be inspected at the *Data used* tab of the explorer, while it is worth mentioning that the default DEA thresholds are easily tunable to meet all users’ expectations.

### Gene co-expression

Gene co-expression analysis has been used successfully in the past for regulatory targets prediction in IPF^9^. Motivated by those previous attempts and in order to complement Fibromine’s PPI network creation tool, we have designed *Gene co-expression* tab to host a human (*IPF_vs_Ctrl*) and a mouse (*BleomycinD14_vs_Ctrl*) lung-specific gene co-expression network (GCN) (Supplementary Fig. S8). Through this tool, with only the selection of any, potentially, disease driving gene, as pinpointed via Weighted Gene Co-expression Network Analysis (WGCNA), the user can plot interactive GCNs spanning any of the top fibrotic phenotype-related modules. Each network is built on the members of the module where the selected feature belongs. Network nodes represent genes with a module membership (MM) and gene significance (GS) above the 60th percentile of their respective module, while edges represent high confidence gene relationships, with a Topological Overlap Measure (TOM) above the 3rd quartile of the respective module’s distribution. The queried gene is marked in red and it is the only node allowed not to have any edge. Last, both MM and GS thresholds can be tuned via Fibromine to dynamically change the strictness of node selection.

### Single cell data

As mapping of genes at the single cell level through the *Gene expression* tab is limited to visual inspection of feature plots, we designed the *Single cell data* tab in order to provide detailed DEA statistical data from one of the biggest datasets regarding lung fibrosis^13^ (233,638 cells from 10 pulmonary diseases and control donor samples). More specifically, through *Search data* sub-tab the user can access DEA data of the top 2000 most variable genes, examined for differentiating expression levels between each pathology and the control cells. These data are expected to increase resolution of the bulk sequencing data presented at the *Gene explorer* tab, as well as to generalize DEA findings to the greater niche of pulmonary pathologies.

Overall, Fibromine web server constitutes a data mining and integration portal suitable for all users irrespective their computational background. As far we are able to know, its highly-automated exploration features render Fibromine, the first IPF-revolving toolkit able to integrate data across species and experimental designs, map them to the single cell level in a visual and tabular format and to offer a quick and tangible way of transcriptomics data projection to an environment of proteomics intercommunication. Last but not least, two other prominent functionalities of Fibromine is mining and presentation of the most biologically promising lung fibrosis-specific miRNA-mRNA interactions and gene co-expression networks.

### Usage of retrieved individual gene expression patterns for novel hypothesis formation

Fibromine’s *Gene explorer* enables interrogation of single/multiple gene expression motifs (consistently recurring DEA results across same/similar experimental conditions), a very useful feature to validate wet laboratory findings and quickly deepen novel hypotheses formation. For example, following leads from cancer and inflammatory diseases, our laboratory has used a primary version of Fibromine’s *Gene explorer* to discover that based on the three larger IPF_vs_Ctrl bulk lung datasets, *MAP3K8* is down-regulated in IPF patients’ lungs^30^. In addition, inspecting *MAP3K8* in higher resolution data via the explorer’s *Map to single cell data* feature instantly revealed an “enrichment” of gene’s expression in monocytic-lineage cells. These findings were further validated in the bleomycin-induced animal model of pulmonary fibrosis, where genetic deletion of *MAP3K8* was shown to exacerbate the modeled disease^30^. All results impelling us to wet lab examination of *MAP3K8* expression during lung fibrosis can be replicated using the *Example* button of *Gene explorer* and then filtering the DEG statistics table for lung tissue and IPF_vs_Ctrl comparisons (Supplementary Fig. S6).

### Identification of “bona fide” differentially expressed genes in human and mice fibrotic lungs

**Figure 5:**
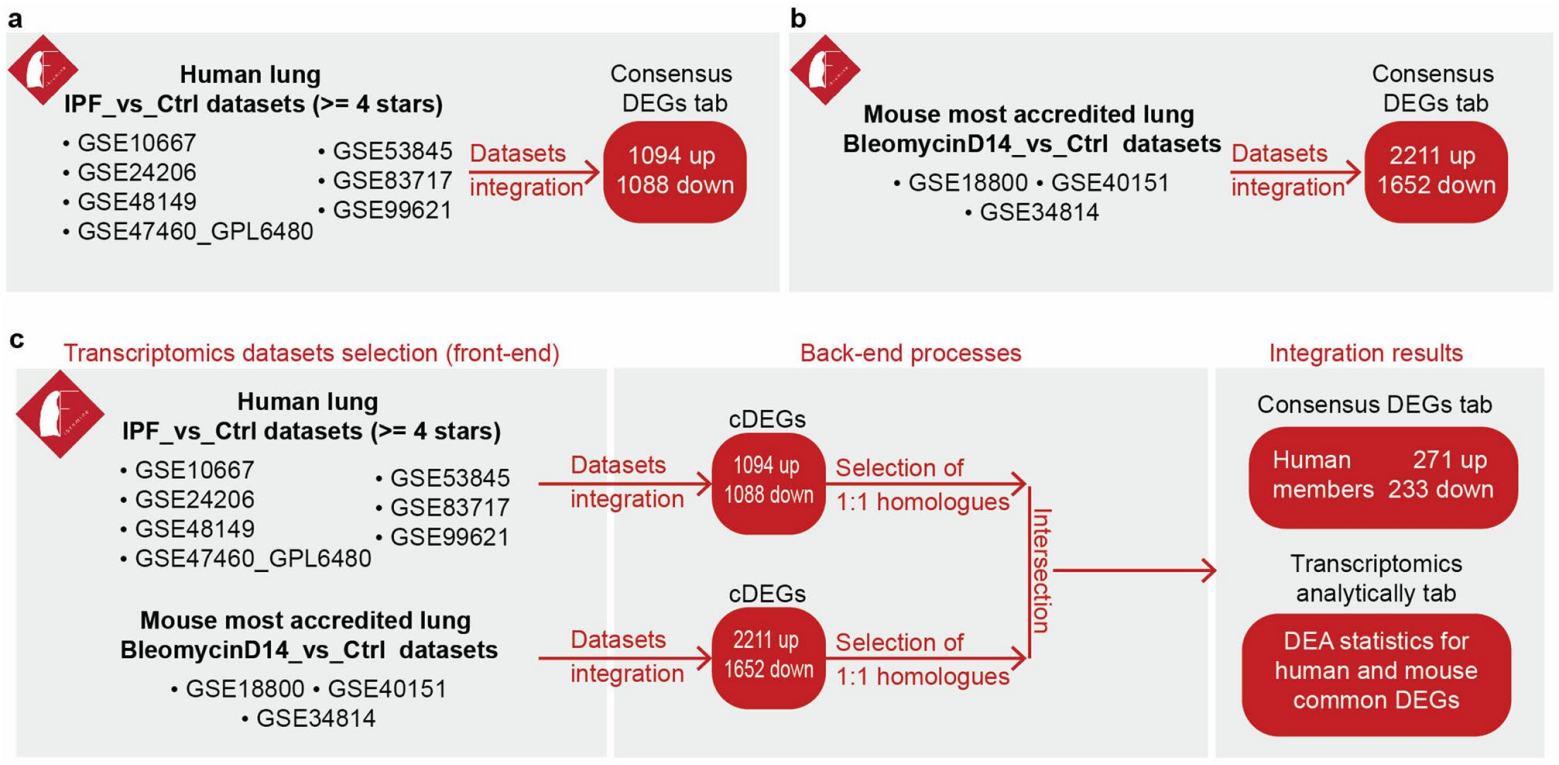
*IPF_vs_Ctrl* lung and *BleomycinD14_vs_Ctrl* datasets integration. **(a)** Integration of the *IPF_vs_Ctrl* lung datasets with at least 4 accreditation stars. **(b)** Integration of the top 3 starred *BleomycinD14_vs_Ctrl* datasets. **(c)** Across-species integration of the (**a,b**) datasets. Selection of the top accredited human and mouse transcriptomics datasets for integration leads to the identification of 504 consensus DEGs. All intermediate and integration output lists can be retrieved through Fibromine. Integration was performed using default differential expression thresholds. *cDEGs:* consensus differentially expressed genes, *DEA:* differential expression analysis. Figure was created using https://www.google.com/slides/about/.

Another very useful function of Fibromine’s web server is its ability to encapsulate differential gene expression data from a great number of samples and datasets sharing similar experimental parameters. This feature is particularly important as it extends over the limited sample size of single datasets and yields results reported by several research efforts. *IPF_vs_Ctrl* and *BleomycinD14_vs_Ctrl* human and mouse lungs respectively, constitute the two most numerous of the 42 unique differential expression comparisons available through Fibromine. To retrieve a list of potential fibrosis drivers for each of these experimental settings, we have initially pinpointed through *Datasets benchmarking* tab (Fig. 1b) those *IPF_vs_Ctrl* human lung datasets with at least 4 stars (Fig. 5a) and the three top accredited *BleomycinD14_vs_Ctrl* lung murine datasets (Fig. 5b). Then, we integrated via *Dataset explorer* the human datasets retrieving a list of 2182 human cDEGs, prior to mouse datasets separate integration which displayed a longer collection of 3863 cDEGs. Both lists can be exported through Fibromine online application using the default thresholds (|FC|> 1.2 and p-value < 0.05) and requiring as input only the selection of the respective datasets from *Transcriptomics datasets* tab main interactive table. For more details on back-end processes, please, refer to respective sections of the publication.

### Across-species datasets integration

Bleomycin-induced pulmonary fibrosis in mouse is a well-established and broadly used model of pulmonary fibrosis^31^ and thus it is expected to bear common ground with the human disease. The aforementioned lists of consensus differentially expressed genomic features provide the opportunity for an inspection of model’s fitness to simulate human pathology, but such a comparative undertaking surely requires extra manual processing. To avoid such a limitation, Fibromine enables automatic between species integration of datasets upon their simultaneous selection through the explorer’s interactive table (Supplementary Fig. S5). As can be seen from Fig. 5c, approximately one quarter of the initial 2182 human cDEGs were found to have a consistently deregulated mouse homologue, with their 1:1 across species homology being an extra factor advocating for a more probable cross-species direct relationship at the molecular level. Cross-species cDEGs can be recreated through Fibromine via integration of the aforementioned datasets.

## Discussion

As more and more omics datasets addressing divergence from steady state emerge and accrue to the existing ones, a big bet for the contemporary biomedical sciences is to be able to fruitfully mine latent information from these already available wealthy data sources in order to guide, accelerate and validate wet laboratory research. This strategy can prove extremely useful especially for rare diseases such as IPF, which logically attract less attention than more prevalent ones. Moreover, biomedical sciences have nowadays entered an era of single cell centred research, enabling pathologies exploration at an unprecedented level of resolution. Nevertheless, pulmonary fibrosis research still lacks a central resource that would help scientists to exploit the vast legacy of bulk omics and single cell datasets and their inherent characteristic such as a smaller drop-out effect^32^. Furthermore, as deconvolution methods have proven to be affected by data pre-processing^33^ steps, a comprehensive collection of consistently handled bulk data could also facilitate the verification of single cell level observations by providing the bulk sequencing material necessary for such analyses.

Taking all the above into consideration, we have developed Fibromine, a database of IPF omics datasets of both transcriptomic and proteomic nature, spanning human and murine research deliverables of various designs. For this purpose we initially manually selected, consistently re-analyzed and carefully curated a great number of bulk transcriptomic and proteomic datasets which consist the backbone of our database. The latter, also includes wealthy third-party annotation and dataset meta-data yielded from literature. Smooth access to Fibromine is ensured by a Shiny-powered web server that simultaneously examines data from multiple complementary perspectives: from a dataset, a single gene/protein, a gene co-expression and protein–protein interaction point-of-view. Crucially, bulk sequencing resolution is increased via provision of in-house single cell level data organized in a tabular format and dynamical connection to external single cell data resources for visual examination. Last, for those users who wish to perform their own downstream analysis, our server supports download of all included data and integration/mining results.

One of the main assets of the hereby presented project is the freedom it provides to the user to fine-tune crucial exploration and integration parameters. To begin with, the user can experiment changing the default DEA thresholds which in turn affect, amongst others, the features of dataset integration and PPI network annotation. In addition, by consulting the available datasets meta-data, the gene/protein annotation included, and more importantly our transcriptomic datasets accreditation system, the user has a quick and tangible means of shaping a well calculated decision regarding which data are contextually best to investigate. Most importantly, thanks to our datasets benchmarking system, the user has a tangible means of comparing transcriptomic datasets based on their otherwise not readily accessible characteristics.

Apart from the well-established data analysis methods used, the operation of Fibromine website is subject to certain assumptions. First, instead on inspecting corrected p-values for cDEG identification, Fibromine uses their nominal counterparts in order to avoid exclusion of borderline significant features, which may be however reported as deregulated using the looser uncorrected threshold in multiple of the user integrated datasets, thus indicating a latent biologically significant feature. In parallel, as multiple testing bias indisputably needs to be taken into consideration, corrected per-dataset statistics for the features reported are presented in the *Transcriptomics* or *Proteomics analytically* tabs. We strongly encourage consultation of the latter prior to any wet or dry laboratory downstream procedure. Furthermore, although very informative, some of the metrics used during datasets benchmarking are based on some general assumptions, such as that the murine homologues of human pro-fibrotic genes exert the same effect in the mouse IPF model and thus, may require future fine-tuning. As far as PPI networks annotation is concerned, the decision of using transcriptomic instead of proteomic data was determined by: (i) the tremendously smaller number of supported DE proteins (693 unique ones) compared to the number of described DE genes (17,153 unique DE protein coding genes; |FC|≥ 1.2 and FDR corrected p-value < 0.05) and (ii) the currently limited number of included proteomic data experimental designs compared to those of transcriptomics nature. Therefore, use of the former instead of the latter would have surely limited PPI annotation options and would have led to the characterization of an excess of nodes as *Non-DE*, thus rendering the tool rather impractical.

Further work needs to be done for Fibromine’s active maintenance and expansion. Despite the careful literature inspection, some of the latest transcriptomic datasets may not have been included into our database and some minor DE comparisons supported by the currently included datasets have not yet been examined. On top of that, our database currently addresses an important yet incomplete portion of proteomic data (*IPF_vs_Ctrl* human datasets) which will also be expanded to include invaluable datasets such as Ref.^34^. Last but not least, enrichment of Fibromine with datasets from other omics technologies such as metabolomics, lipidomics and predominantly a greater number of single cell datasets are some of our top priorities.

Conclusively, this endeavour set out to implement from scratch a centralized web resource for the acceleration of IPF research. Through Fibromine, both computational and most importantly non-computational background supported biomedical scientists can quickly and effortlessly obtain, integrate and compare information regarding DE events during a wide variety of IPF-related conditions. Hopefully, Fibromine will prove itself the driving force for novel hypothesis formation and new biomarkers discovery.

## Methods

### Datasets re-analysis and curation

Microarray datasets re-analysis was performed using ad hoc developed pipelines based on well-established tools and methods, while metaseqR2 Bioconductor package^35^ was used for the re-analysis of RNA-seq data.

For microarrays data processing, limma^36^, oligo^37^ and beadarray^38^ Bioconductor packages consisted the analysis workhorses while arrayQualityMetrics package was utilized for quality control purposes^39^. More specifically, probe intensities were within-technology consistently background corrected, within- and across-technologies uniformly normalized using quantile normalization and then summarized to the gene level using a weighted average. Control sequences and probes mapping to multiple HGNC gene symbols were removed from further analysis. DEA was conducted for all datasets using the limma moderated t-test statistics method.

RNA-seq fastq files were mapped to GRCh38.p13 and GRCm38.p6 genomes using a two steps alignment pipeline that exploits HISAT2^40^ and Bowtie2^41^ aligners. Initially, HISAT2 was used to map reads in a splice aware fashion, while those failing to align were delivered to Bowtie2 for a second, more sensitive alignment round. Further processing was conducted using the Bioconductor package metaseqR2^35^: raw counts files were quantified at the gene level, normalized with EDASeq^42^, filtered using default parameters and statistically analysed for DE using PANDORA algorithm. The latter combined the results of the well-known and broadly used DESeq^43^, DESeq2^44^, edgeR^45^, limma-voom^36^ and ABSSeq^46^ DEA methods, leading to more precise lists of differentially expressed genomic features. More precisely, PANDORA combines individual DEA algorithms p-values in a weighted manner, with weights shaped according to each method’s performance during real-data based simulations. As a result, among others, PANDORA achieves a better precision-recall trade-off and reduces the effects of gene length in downstream analyses, such as pathway analysis.

For microarray and RNA-seq datasets, sample outliers were identified prior to removal using PCA and MDS plots, respectively, as well as samples hierarchical clustering of log2-scaled normalized values of the thousand top DE genes. The later were defined using the thresholds of |FC|> 1.2 and a significant p-value at an FDR threshold of 5%.

Proteomics data were retrieved directly from published tables and DE thresholds were kept *as is* in each original publication. An exception was made for a single dataset^47^ due to the very limited number of DE proteins returned otherwise. Proteins quantified in mixtures/same aptamer were removed.

### Identification of consensus differentially expressed features

During same species datasets DEA results integration, a feature (either transcript or protein) is called consensus differentially expressed if it has been found consistently deregulated (in the same direction of deregulation) in at least half of the user-selected datasets and not significantly deregulated towards the opposite direction in any of the rest; *Consensus DEGs/Consensus DEPs* tabs. According to those, we report as consensus direction of deregulation, the one most frequently encountered across them, while as consensus fold change the mean of their fold change values. When integrating datasets from multiple species, a feature is defined as consensus DE if it has 1:1 human:mouse homology (based on Ensembl data) and has been found consistently deregulated in at least half of each species’ selected datasets. Datasets examining explicitly non-coding genes are not considered for across species integration. Consensus fold change is calculated as in the case of same species data. For reasons of clarity, only the human component of across species datasets integration is summarized in *Datasets explorer’s Consensus DEG* and *Consensus DEPs coded* tabs. In both the above cases, DEA thresholds are |FC|> 1.2 and p-value < 0.05 for genes and those applied during original analysis for proteins. For more on hypothesis testing thresholds selection, please, refer to “Discussion” section of the paper. All thresholds for DE genes identification are user-tunable from within Fibromine.

### Pathway analysis

Fibromine’s *Dataset explorer* supports pathway analysis of cDEGs. Specifically, enrichR package is used to connect with the Enrichr database and perform over-representation analysis for up and down regulated genomic features separately, based on five libraries: KEGG, BP GO, MF GO, BP GO and one of COVID-19-related associations. For more on current use and implementation of the aforementioned gene set libraries please refer to the latest Enrichr publication^48^.

### Heatmap and volcano plot creation

Dataset-specific Fibromine’s interactive heatmaps and volcano plots are crafted using heatmaply^49^ and plotly R package^50^, respectively. Clustering of each dataset’s samples is performed on z-score scaled log2-transformed normalized expression values of the top one thousand DE genes using Euclidean distance and complete linkage for clusters comparison. Volcano plots specify deregulated genes based on the thresholds of |FC|> 1.2 and p-value < 0.05. A significant, yet non-comprehensive list of genes known to be implicated in pulmonary fibrosis are indicated in every volcano plot.

### Condition-specific protein–protein interaction networks creation

PPI networks are shaped according to Fibromine-incorporated STRING database^18^ data. More specifically, each network consists of two interaction shells, with the first including maximum nine, high confidence (interaction score > 700) proteins interacting with the queried one. For the determination of second shell elements, the two most confident interactors for each of the first shell proteins are selected. Proteins already-considered for the first shell creation are not considered for the second one. All networks have a DrL layout with protein interaction scores used as edge weights.

For the condition-specific annotation of networks, genomic features coding for the network elements are identified and their differential gene expression statistics are recovered from the datasets corresponding to user-selected experimental parameters. From the aforementioned genes only those corresponding to UniProt/Swiss-Prot members are retained and for the cases of 1:many UniProt/Swiss-Prot:gene entries the gene featured by UniProt as primary is utilized. Afterwards, each network node is assigned a label of a consensus direction of deregulation: “Unknown” if the gene product is not a Swiss-Prot member, “Non DE” if the corresponding gene is not DE and “Upregulated” or “Downregulated” for the deregulation cases. The latter two labels are shaped according to the following rules: if a gene is found DE in a single dataset or consistently deregulated in a couple of datasets, its direction of deregulation is kept *as is*; if a gene is found DE in a couple of datasets with an inconsistent direction of deregulation between the two, the biggest dataset’s direction of deregulation is maintained; if a gene is found DE in multiple datasets, then the most frequent direction is utilized. By default, DE genes are defined as those having an absolute fold change bigger than 1.2 and a p-value smaller than 0.05. Multiple testing corrected statistics are presented at the *Data used* tab of the explorer.

### Gene co-expression networks creation

For the human network, *IPF_vs_Ctrl* lung tissue datasets with more than four stars assigned were selected (GSE10667, GSE24206, GSE48149, GSE47460_GPL6480, GSE53845, GSE83717, GSE99621), while the three most accredited *BleomycinD14_vs_Ctrl* were chosen for the mouse one (GSE18800, GSE40151, GSE34814). Normalized, z-transformed gene expression values were applied to determine the scale-free co-expression modules via WGCNA^51^. Biweight midcorrelation, a more robust alternative of Pearson coefficient, was used to create a signed network, while signed Topological Overlap Measure (TOM) was calculated by adjacency transformation to decrease noise. Network modules were identified using (1-TOM) as a distance metric and module eigengene (ME) was determined using default parameters. Closely clustered modules (0.25 and 0.40 distance determined by hierarchical clustering for human and mouse, respectively) were merged. MEs were correlated to the trait of interest (fibrotic/non-fibrotic tissue) and those with a significant Pearson correlation coefficient were maintained (|ρ|> 0.6; p-value < 0.05). Potential phenotype-drivers were pinpointed by intra-modular analysis having a statistically significant module membership and gene significance value (p-value < 0.05). Pearson co-efficient for the latter two metrics can be adjusted via Fibromine server for a more thorough and objective features selection.

For the creation of network visualisations, all features of the selected gene’s module with module membership and gene significance above the (default) 60th percentile are selected. Network edges represent correlations in the 3rd quartile of the pre-calculated TOM and with the exception of the queried gene, all genes having a zero degree of connectivity are dropped prior to minimum spanning tree calculation and network visualization. Network layout is automatically chosen and the thresholds of module membership and gene significance are user tunable.

### Single cell data analysis

Seurat package v.4 was used for the analysis of single cell data found here. In detail, “empty” cells, as annotated during original analysis, were removed prior to identification of the top 2000 most variable genes using the *vst* method (FindVariableFeatures). Finally, Wilcoxon rank sum test was used for DEA of the above mentioned genes using default parameters (FindMarkers).

## Supplementary Information

Supplementary Information.

## Data Availability

The data that support this study can be accessed freely via http://www.fibromine.com/Fibromine, a Shiny-based web tool.

## Code availability

The code for the creation and operation of Fibromine web server is available via https://github.com/dfanidis/Fibromine.
