# Supplementary material for: Fibromine is a multi-omics database and mining tool for target discovery in pulmonary fibrosis
Source: Sci Rep. 2021 Nov 5;11:21712. doi: 10.1038/s41598-021-01069-w (PMC8571330; doi:10.1038/s41598-021-01069-w)
Supplement: Supplementary file 1 — Supplementary Information. [file 41598_2021_1069_MOESM1_ESM.pdf]

Supplementary information

Fibromine is a multi-omics database and mining tool for target discovery in pulmonary fibrosis

Dionysios Fanidis, Panagiotis Moulos and Vassilis Aidinis

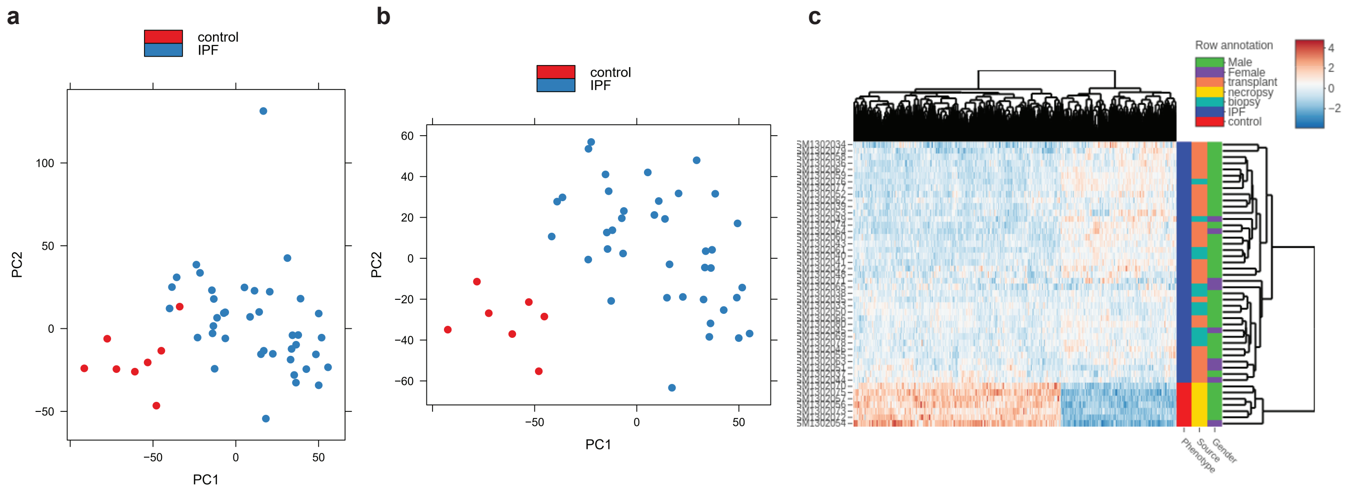

Supplementary Figure 1: Datasets manual curation example. All transcriptomic datasets included into Fibromine were manually curated using PCA/MDS and samples hierarchical clustering plots. **(a)** PCA plot of the GSE53845 dataset prior to manual curation. **(b-c)** PCA and hierarchical clustering plot of the same dataset post to manual curation. *PCA: principal component analysis; MDS: multi-dimensional scaling. Figure created using ggplot2 v3.3.5, heatmaply v1.2.1 and Google slides.*

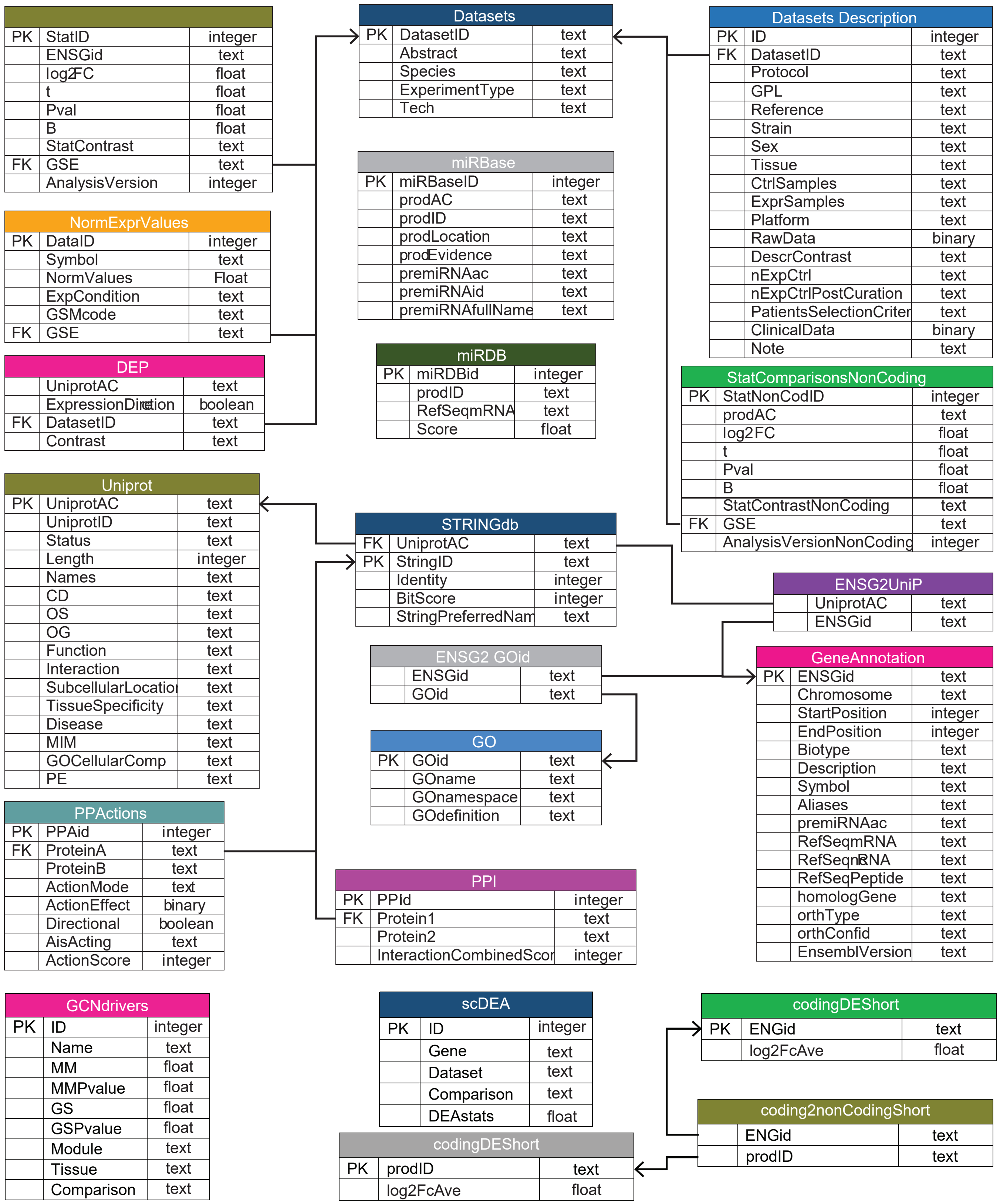

Supplementary Figure S2: Fibromine schema. *Figure created using Google slides.*

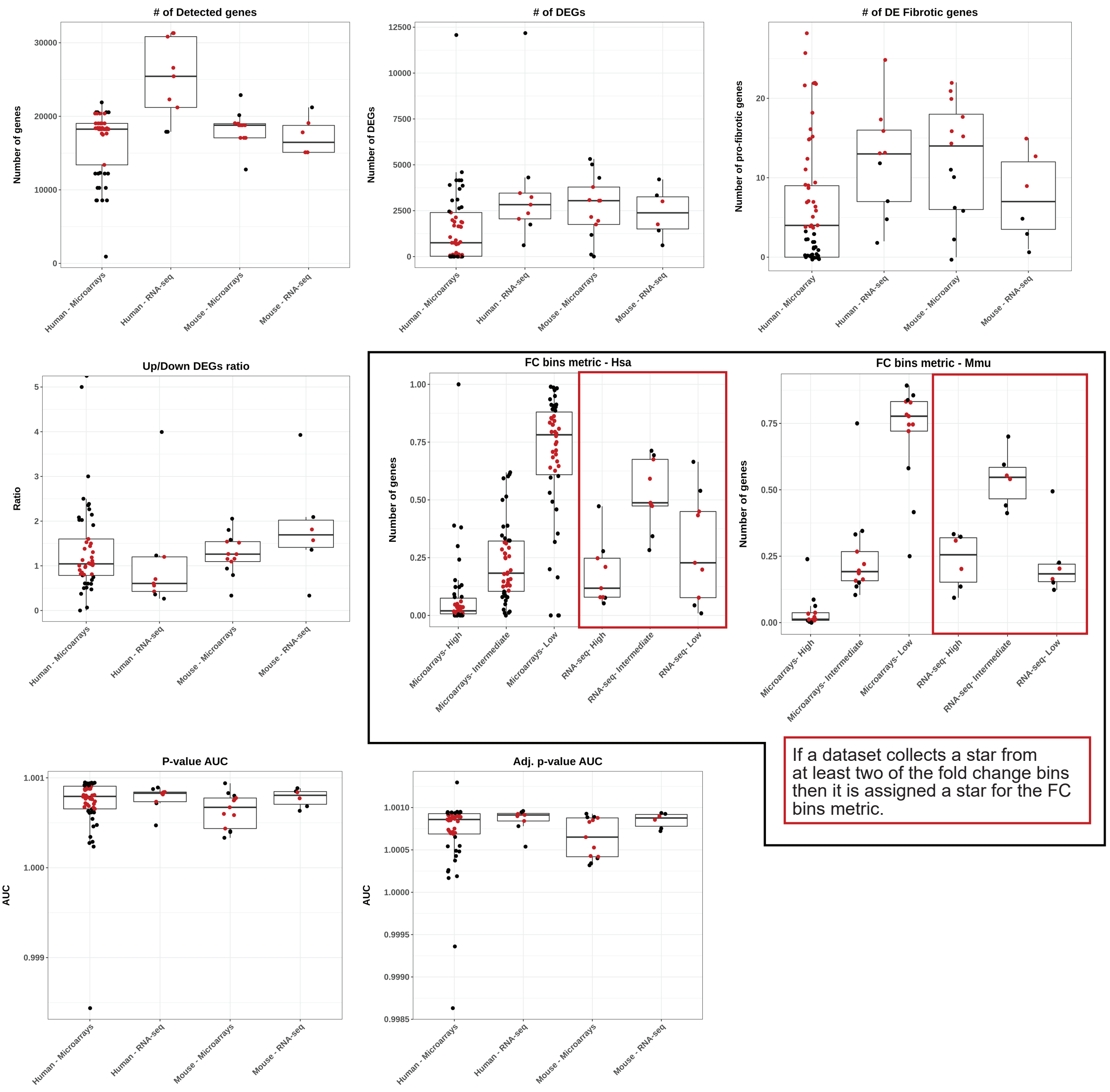

Supplementary Figure S3: Fibromine datasets benchmarking analytically. All coding transcriptomic datasets were assessed using seven metrics in an attempt to help the user choose those of interest. After separating datasets/comparisons as depicted in Figure 2a, we have created a per-metric distribution for each of the dataset groups. A dataset was starred for a certain metric (red dots), if the latter's value was within the interquartile range of the respective distribution, as depicted above. Exception to the latter is the Detected genes and Pro-fibrotic genes metrics, where the median to 90th percentile and the above the median ranges were utilized. ● *Starred dataset/comparison* ● *Non-starred dataset/comparison. Figure created using ggplot2 v3.3.5 and Google slides.*

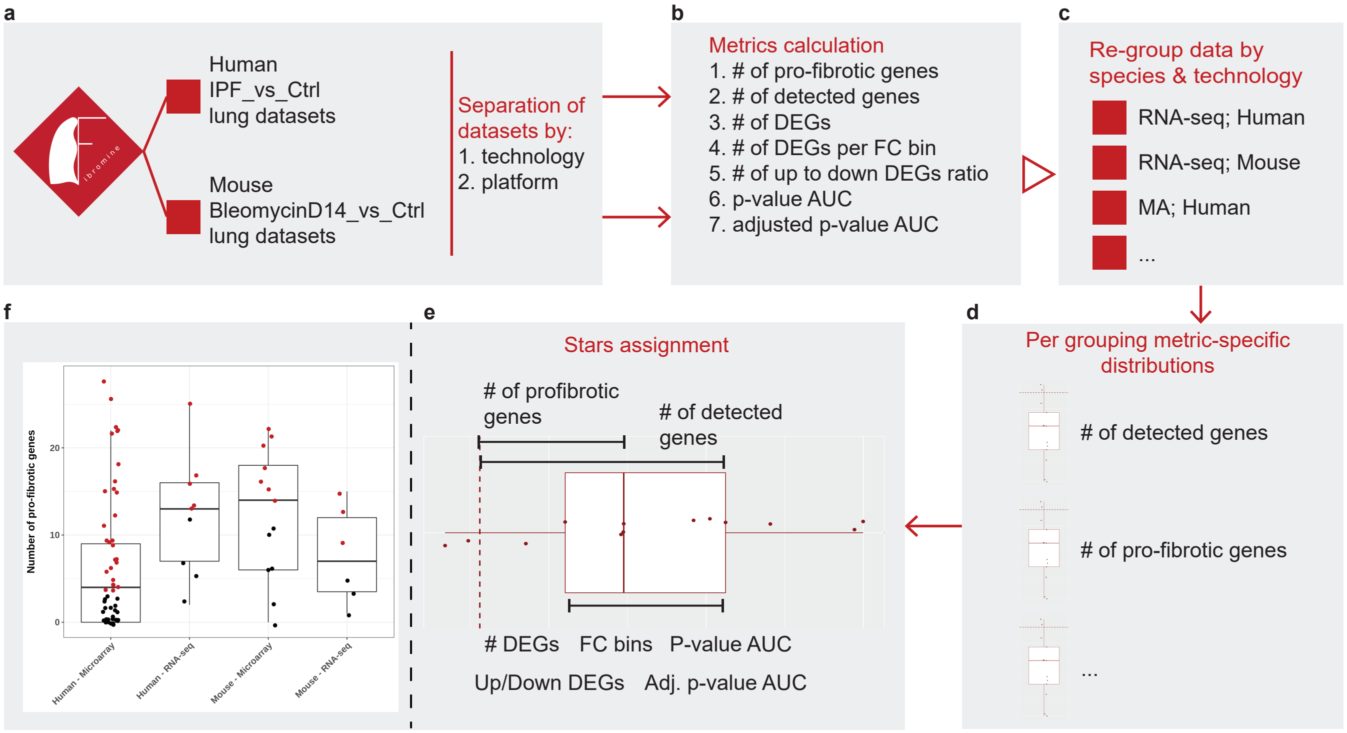

Supplementary Figure S4. Datasets benchmarking for major Fibromine DEA comparisons. In order to reveal the most homogeneous transcriptomic datasets for two major Fibromine DEA comparisons **(a)** seven metrics were calculated **(b)**. Then, data were re-grouped per species and technology **(c)** and metric-specific distributions were created for each of the final groupings **(d)**. Last, every dataset was evaluated with respect to its group and assigned a per-metric star if the calculated criterion value lied within a pre-specified interval of the respective distribution **(e)**. Each dataset received at maximum seven and at minimum zero stars. Datasets with many stars are more closely related to each other than to the rest of the group. **(f)** A real example of stars assignment: red dots are datasets starred for the *Number of pro-fibrotic genes* criterion. Boxplots depict the interquartile range and median of the data; whiskers extend no longer than 1.5 times the length of the boxplot. *DEGs: differentially expressed genes; FC: fold change; AUC: area under the curve. Figure created using ggplot2 v3.3.5 and Google slides.*

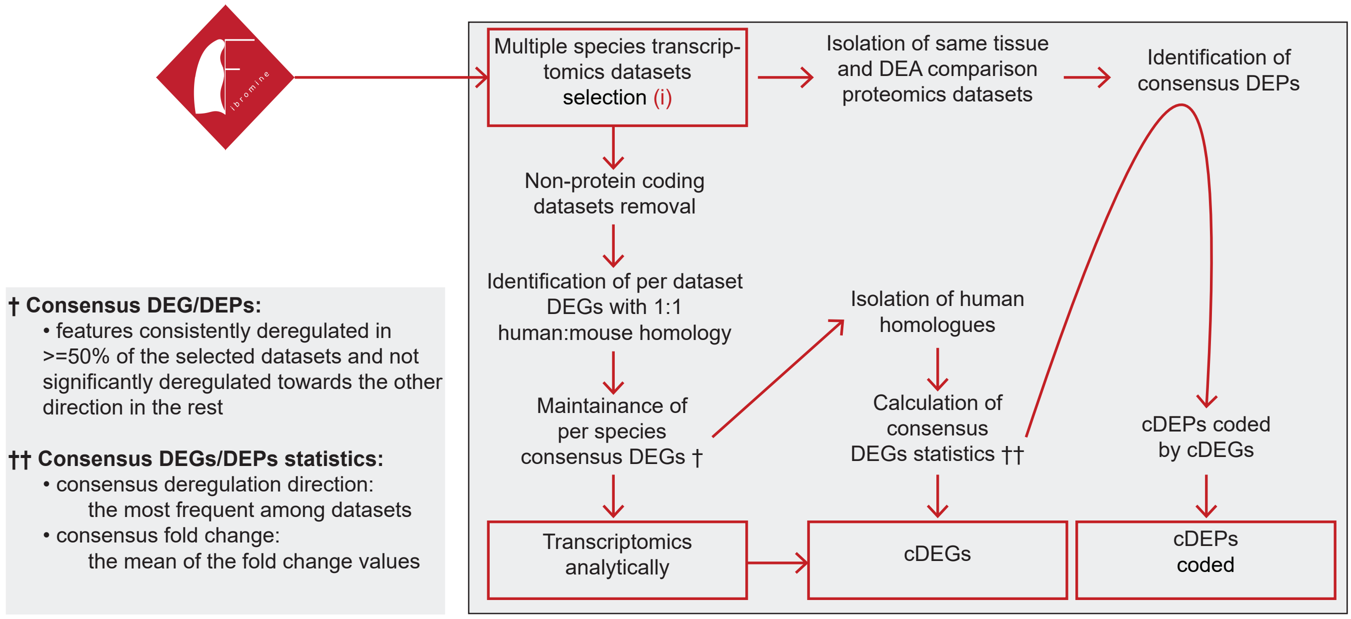

Supplementary Figure S5. Across species datasets integration workflow. Across species transcriptomic datasets *Dataset explorer* back-end integration workflow. If datasets from more than one species is selected, then datasets integration workflow is slightly modified and finally, for clarity reasons, focus is given to human consensus DEGs. Consensus DEPs returned must share the same direction of deregulation with their coding gene. Boxes of red correspond to front-end elements, while the rest account for back-end processes. *cDEGs: consensus differentially expressed genes; cDEPs: consensus differentially expressed proteins. Figure created using Google slides.*

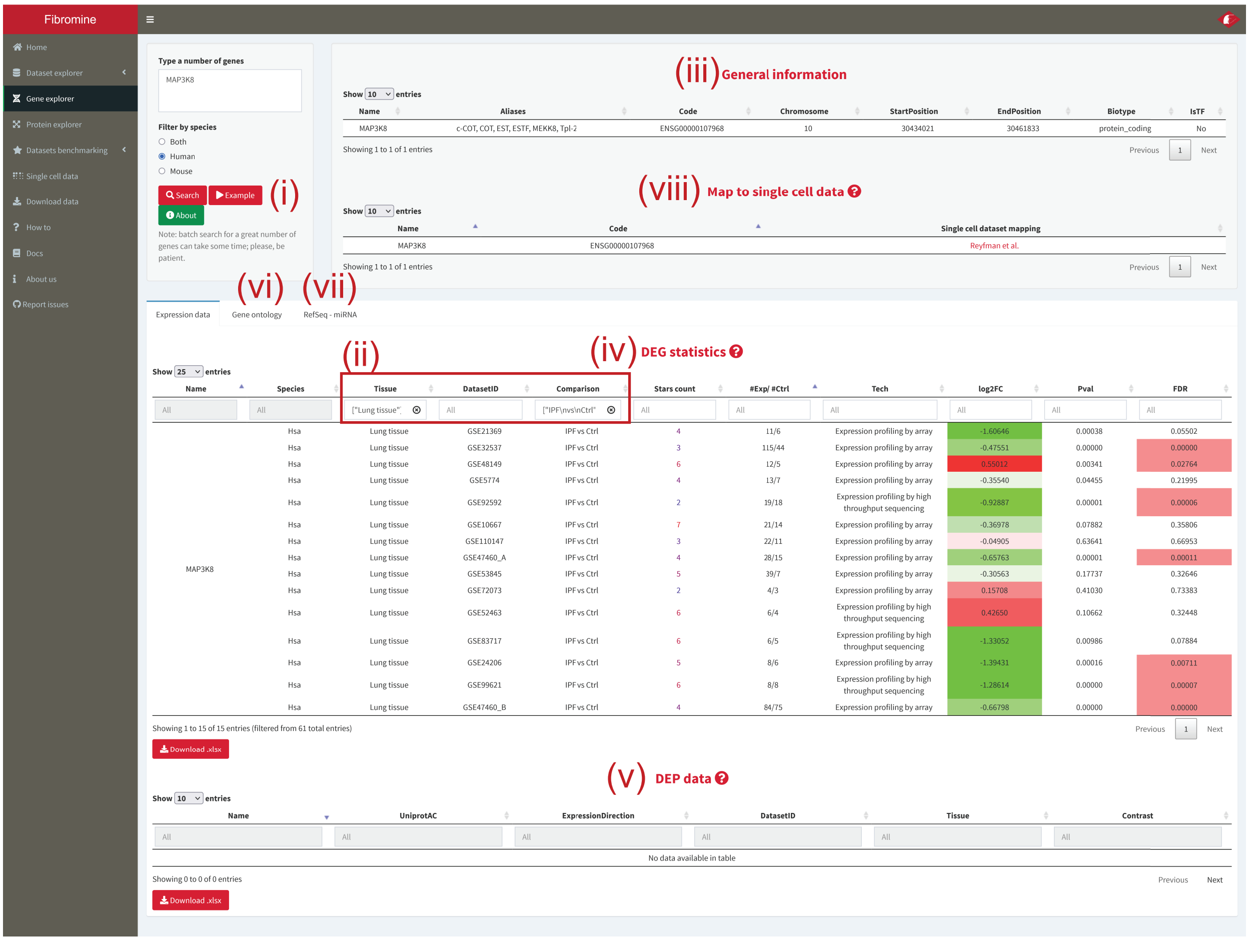

Supplementary Figure S6. Gene explorer interface example. Through Gene explorer the user can interrogate Fibromine data for one or multiple genes of interest simultaneously. Here the output of the *Example* button (i) is displayed. *DEG statistics* table results are filtered to present lung tissue IPF_vs_Ctrl comparisons (ii). Initially, some general information is displayed (iii), while DEG statistics table (iv) records in detail the respective expression patterns. Differentially expression analysis data from the proteomic datasets corresponding to the genomic feature queried are presented at the DEP data table (v), if any. Gene ontology (vi) and RefSeq - miRNA tabs (vii) present the GO terms matched with the query elements, RefSeq data and miRNA-mRNA potential interactions sourced from miRDB, respectively. Finally, the user has the option of exploring gene expression at the single cell level via dynamic redirection to the NU-Pulmonary online resource (viii) in a species-specific manner. *Figure created using Google slides.*

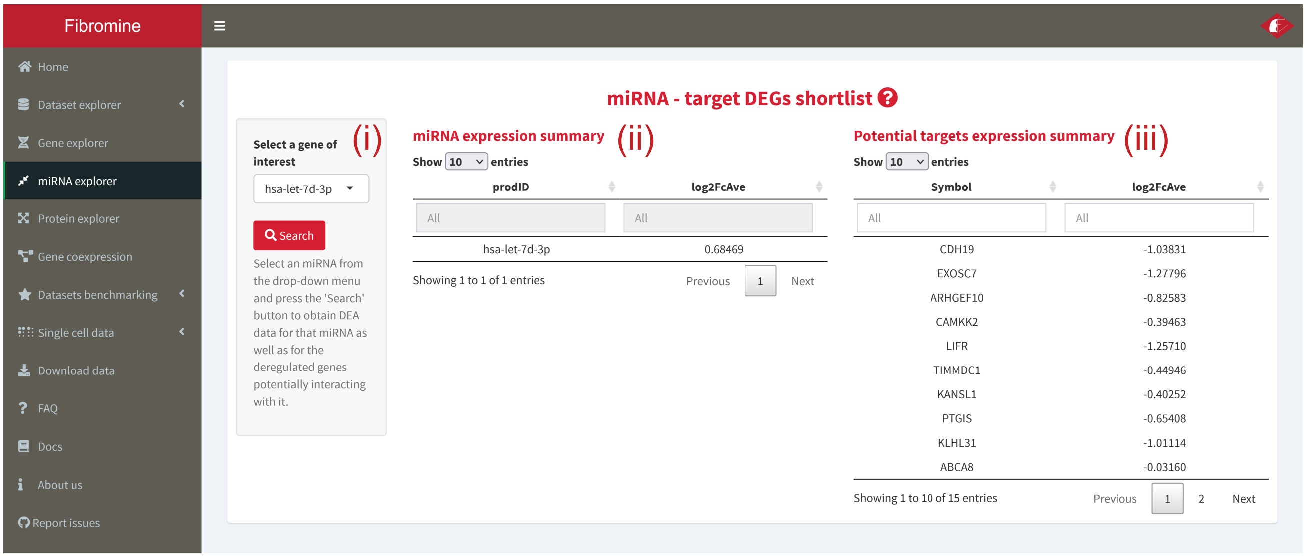

Supplementary Figure S7. miRNA explorer interface example. miRNA explorer holds all miRNAs consistently differentially expressed (DE) in non-coding array *IPF_vs_Ctrl* lung datasets along with their target mRNAs found deregulated under the same experimental conditions. With the selection of a non-coding RNA (i) and press of the "Search" button, the user obtains integrated DE data for the queried miRNA (ii) and the respective statistics for all its targets characterised by the opposite direction of deregulation (iii). *Figure created using Google slides.*

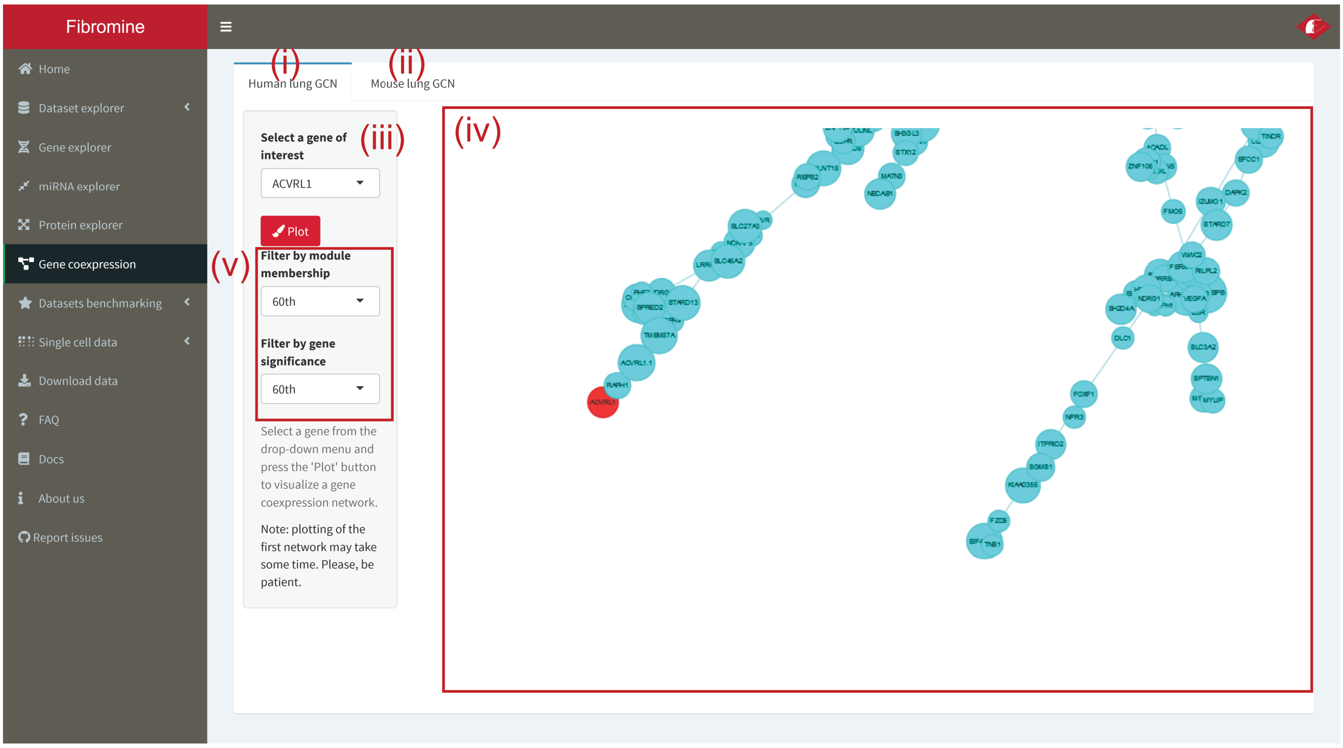

Supplementary Figure S8. Gene coexpression interface example. Via Gene coexpression tab, the user can visually explore genes of important co-expression modules from a human *IPF_vs_Ctrl* (i) and a mouse *BleomycinD14_vs_Ctrl* lung dataset (ii). By selecting any gene from the drop-down list of the tool (iii) and pressing the "Plot" button, a gene co-expression network based on queried gene's module is created (iv – network zoomed in; queried gene in red). Module members selected for depiction are those with module membership and gene significance values above, by default, the 60th percentile of the respective distribution. The thresholds can be user-tuned from the panel on the left (v). Network edges represent a Topological Overlap Measurement (TOM) above the 3rd quartile of the module TOM values and all nodes, but the queried gene, are not allowed to have no edges. *Figure created using Google slides.*

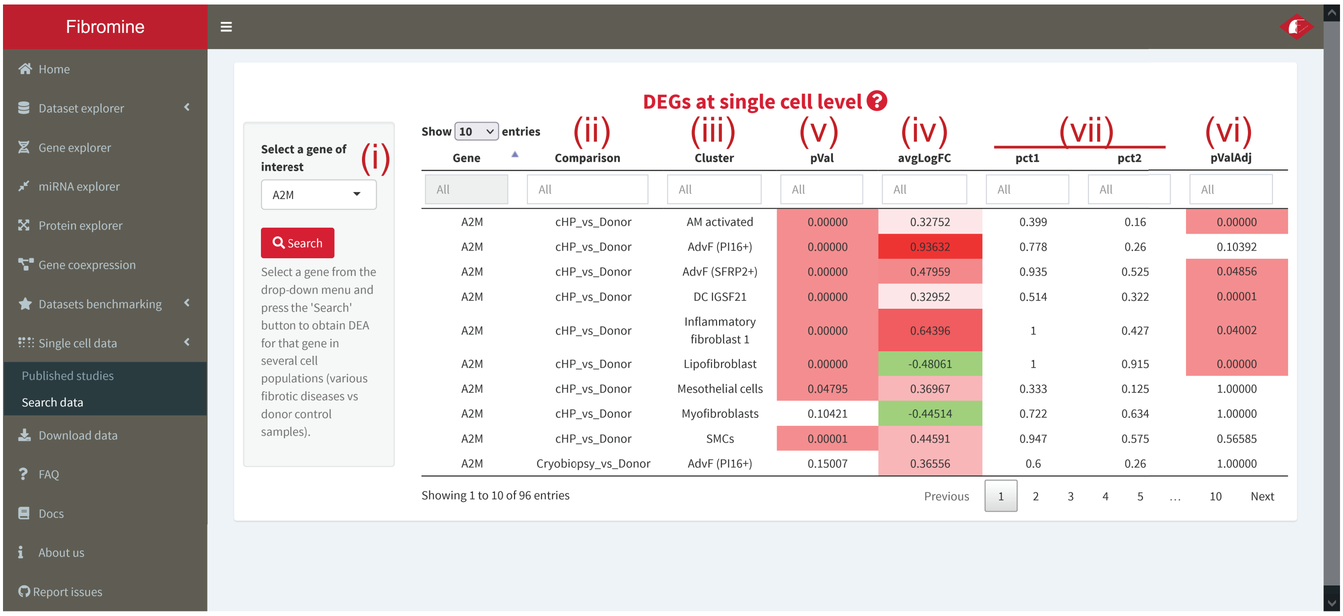

Supplementary Figure S9. Single cell data exploration interface example. Differential expression analysis results from the single cell dataset of Mayr et al. (2021) can be investigated via *Single cell data > Search data* tab of Fibromine. The user can query the expression profile of the most variable features (i) as it changes between any of the ten included pathologies relative to control donor samples (ii) in a set of 45 cellular types (iii). Direction of deregulation is color coded in the Average log Fold Change (avgLogFC; iv) column: positive fold changes are marked with shades of red and negative fold changes with shades of green. P-value (pVal; v) and p-value adjusted (pValAdj; vi) column cells are colored in red every time the respective value is smaller than 0.05. Columns pct1 and pct2 (vii) record the percentage of cells in each comparison part that expresses the queried gene. *Figure created using Google slides.*
